# The rosette-forming glioneuronal tumor mimicked cerebral cysticercosis: a case report

**DOI:** 10.1007/s10072-021-05199-x

**Published:** 2021-05-20

**Authors:** Dan Zhu, Ailan Cheng, Nickita T. L. Benons, Shuguang Chu

**Affiliations:** 1 Department of Radiology, Shanghai East Hospital, Tongji University, No. 150 Jimo Road, Pudong New Area, Shanghai, 200120 China; 2 Tongji University, No. 1239 Siping Road, Yangpu District, Shanghai, 200092 China

**Keywords:** Rosette-forming glioneuronal tumor, Cerebral cysticercosis, Magnetic resonance imaging, Hemorrhage

## Abstract

**Introduction:**

Rosette-forming glioneuronal tumor (RGNT) is a rare variety of slow growing mixed glioneuronal tumor involving primarily fourth ventricular region. This is a comprehensive analysis of a 22-year-old woman with RGNT composed of mainly cystic components. In addition, the case showed multiple lesions located in brain parenchyma which mimicked cerebral cysticercosis. Here, we analyzed this case and listed some characteristics of RGNTs in reported literature which occurring in atypical locations for further understanding it.

**Case report:**

A 22-year-old woman presented with a history of transient dizziness, nausea, and vomiting. Magnetic resonance imaging (MRI) showed multiple cystic lesions in brain parenchyma and then the patient was diagnosed with cerebral cysticercosis possibility. Empirical anti-infective therapy in addition to a follow-up post 2 weeks of MRI examination showed the lesions unchanged. Finally, a biopsy of the right cerebellar hemisphere lesions verified RGNT.

**Conclusion:**

RGNT is an uncommon tumor classified as grade I glioma by World Health Organization (WHO) with slightly longer course. The imaging findings of RGNT are not specific especially in atypical areas. RGNT is rare, but we should also consider the possibility in diagnosis and differential diagnosis.

## Introduction

The rosette-forming glioneuronal tumor (RGNT) was first described by Komori et al. in 2002. As it was initially thought as dysembryoplastic neuroepithelial tumor (DNT) of the cerebellum [1]. In 2007, it was classified as grade I glioma by World Health Organization (WHO). RGNT occurs most often in young women with mean age of onset at 23.57 years [2]. There are few literatures regarding the imaging features and prognosis of RGNT. For most of the literatures on RGNT are case reports. RGNT is most commonly located in the fourth ventricle; however, recent reports demonstrated that RGNT can also occur at sites outside its usual locations. The lesions are mostly comprised of cystic-solid or solid, and the solid components present heterogenous enhancement. Here, we describe a rare case of a 22-year-old woman with RGNT in bilateral cerebellar hemisphere, brain stem, and left thalamus who was misdiagnosed as cerebral cysticercosis before biopsy.

## Case report

**Fig. 1:**
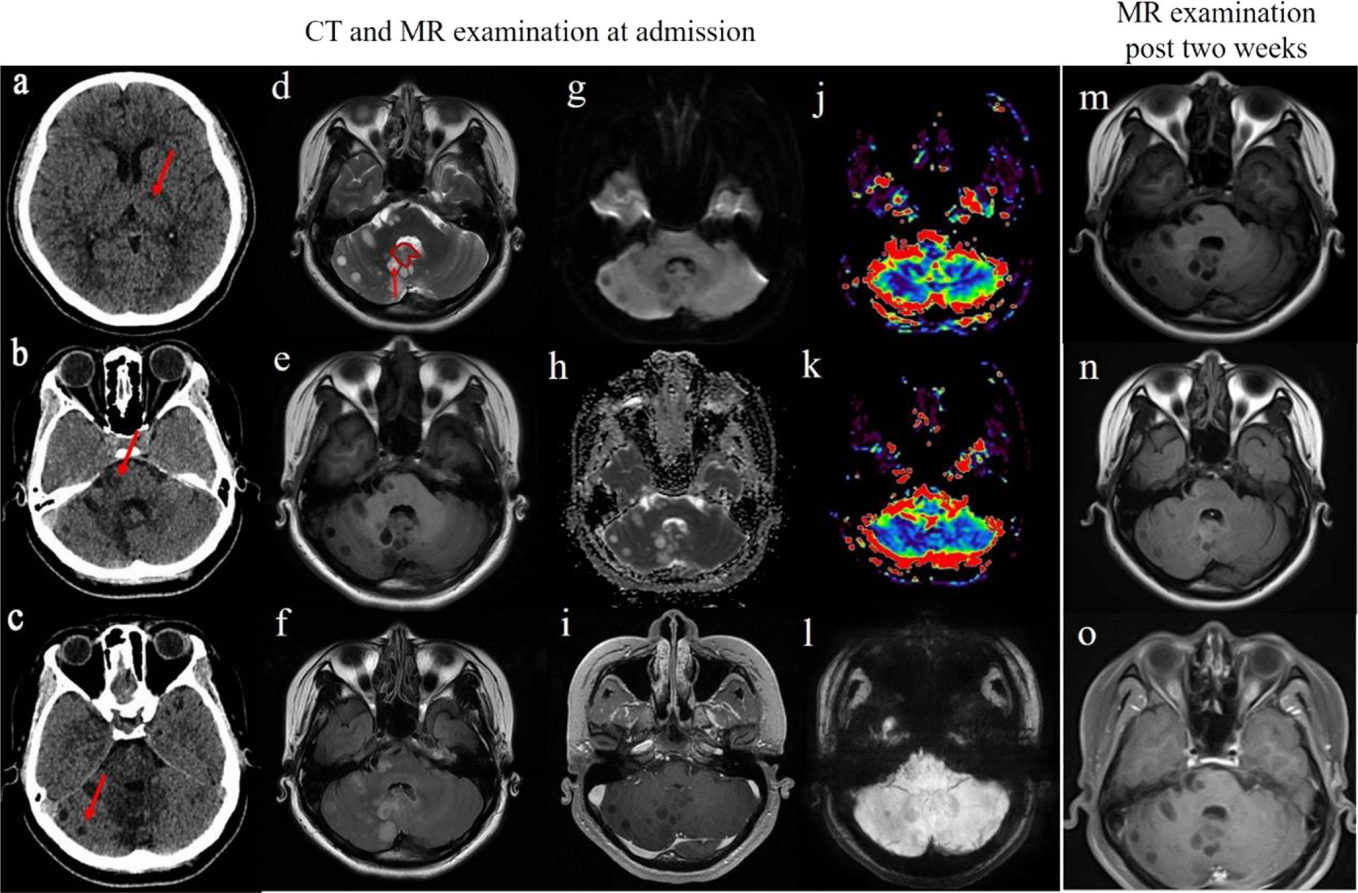
**a**–**c** CT findings show the tumors in left thalamus, brain stem, and bilateral cerebellar hemisphere (red arrow). **d**–**l** MRI findings. The lesions in bilateral cerebellar hemisphere. **d** Hyper-intense in T2-weighted image and small nodule-like higher signal (red arrow) along with circled solid component. **e** Hypo-intense in T1-weighted image. **f** T2 flair image showed iso-hyper intense. **g** Hypo-intense in DWI. **h** Hyper-intense in ADC. **i** No obvious enhancement majority and minority presented as mild annular enhancement. **j**–**k** Perfusion-weighted imaging color map, decreased regional cerebral blood flow, and regional cerebral blood volume. **l** minor hemorrhage in the lesions. **l** SWI showed minor hemorrhage. **m**–**o** MRI findings for post 2 weeks. **m**–**o** Corresponding T1WI, T2 flair, and T1WI enhancement, no obvious changes compared with before

A 22-year-old woman presented with a history of transient dizziness, nausea, and vomiting. No neurological deficits were apparent; however, on further evaluation, initially with computed tomography (CT) scan, revealed multiple cystic hypo-dense mass lesions in bilateral cerebellar hemisphere, brain stem, and left thalamus with unclear boundary (Fig. 1a–c). Magnetic resonance imaging (MRI) confirmed these lesions presented as hyper-intense in axial T2-weighted images and hypo-intense in axial T1-weighted images (Fig. 1d, e). The solid components were visible in some of the lesions in axial T2-weighted images (Fig. 1d). In addition, axial T2 FLAIR revealed iso-hyperintense (Fig. 1f). Diffusion-weighted imaging (DWI) and apparent diffusion coefficient (ADC) showed no restricted diffusion (Fig. 1g, h). After contrast, one of the tumors showed mild peripheral enhancement, while others presented no enhancement (Fig. 1i), and small nodule-like higher signals in T2-weighted images were present (Fig. 1d). While, on perfusion-weighted imaging, the lesions were hypo-perfused (Fig. 1j, k).

**Fig. 2:**
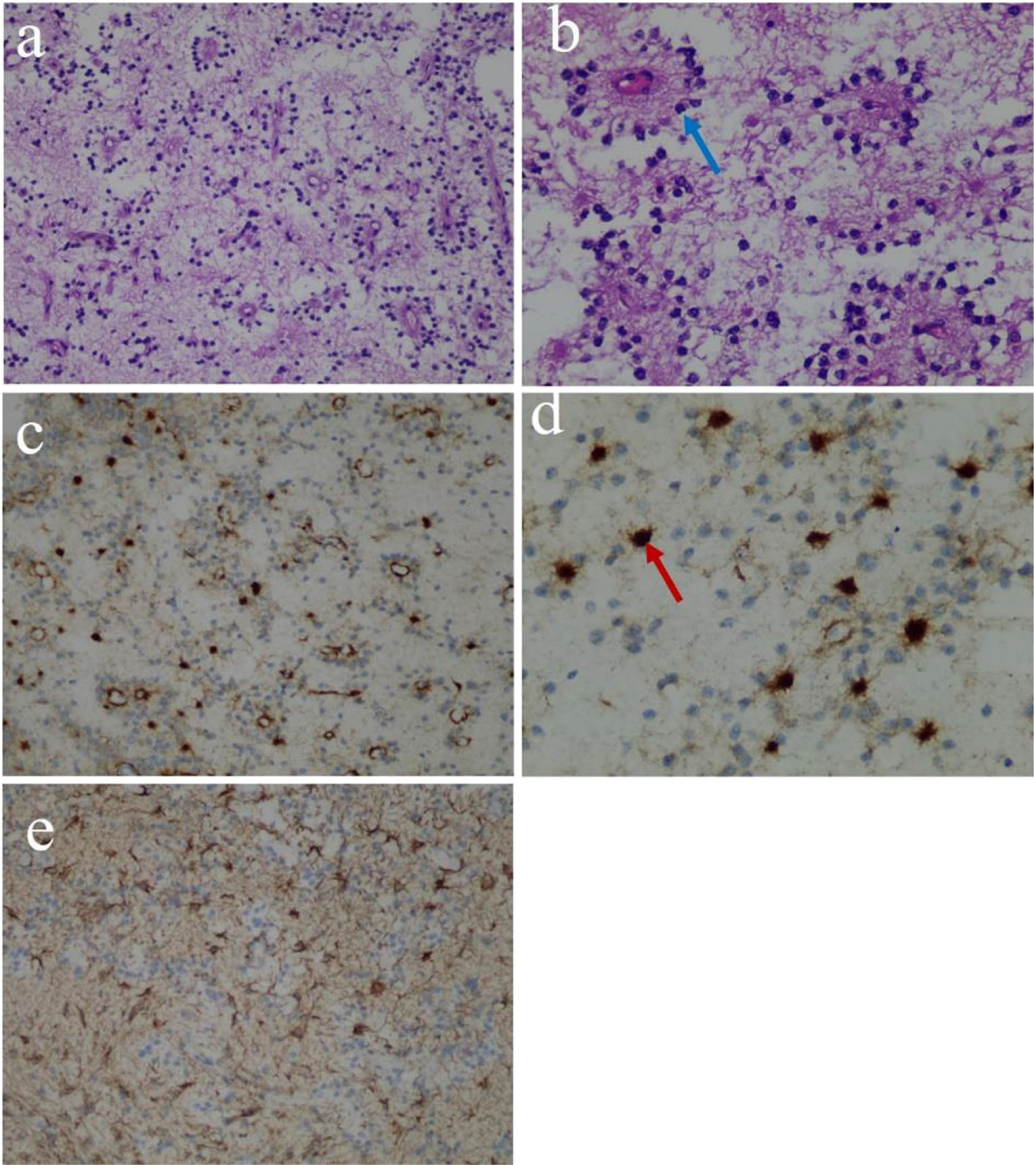
**a**–**d** Pathological pictures of the tumor. **a** Glioneuronal tumor with glial and neurocytic components. **b** Neurocytic rosette: small round nuclear tumor cells distributed in a network and a ring-like array of neurocytic tumor cell nuclei around an eosinophilic neuropil core (blue arrow) (hematoxylin-eosin: a×200; b×400). **c**, **d** Synaptophysin immunopositivity in the pericapillary area of a perivascular pseudorosette (red arrow) (c×200; d×400). Diffuse positivity for glial fibrillary acidic protein (GFAP) in the glial component of the tumor(e×200)

Based on the above radiological findings, the woman was initially diagnosed with cerebral cysticercosis most possibly. Naturally, primary tumors of the central nervous system and metastatic tumors were differential diagnoses. Subsequently, she was admitted to the infectious diseases department to conduct empirical anti-infective therapy in addition to a follow-up post 2 weeks of MRI examination. Over the course of 2 weeks, she underwent further laboratory examinations, including serologies (specifically enzyme-linked immunotransfer blots (EITBs)); however, the results were negative. Meanwhile, eosinophilic cells and lymphocytes were neither found in cerebrospinal fluid (CSF). Other parasite antibodies were not discovered either. Most importantly, there were no significant changes in the MRI findings after 15 days (Fig. 1m–o). Finally, to determine an ultimate diagnosis, she underwent a biopsy of the right cerebellar hemisphere lesions in which the histopathological results confirmed a WHO grade I RGNT ultimately. Microscopically, the tumors showed that small round nuclear tumor cells were distributed in a network and arranged into a chrysanthemum-shaped cluster surrounding the nerve with single permutation. Synaptophysin immunopositivity in the pericapillary area of a perivascular pseudorosette was shown, along with scattered neurons in the focal areas were also visible. In addition, glial fibrillary acidic protein (GFAP) in the astrocytic component of the tumor was diffuse distribution. (Fig. 2a–e).

## Discussion

RGNT is an unusual disease, and it is considered an independent entity of glioma, which is categorized as grade I by World Health Organization (WHO) due to its characteristics of containing both neural and glial components [3]. It is generally considered to be benign, but there have been reports that some can be invasive [4]. The disease was initially thought to only occur in the fourth ventricle, and the typical imaging characteristics are mid-line lesions which appeared in the fourth ventricle and extended to adjacent structures [5]. On MRI, RGNT typical imaging findings are relatively well circumscribed, with both solid and cystic components with T1-hypo-intense and T2-hyper-intense located in or around the fourth ventricle. Gadolinium-based contrast enhancement could show variable or no enhancement, but with increasing reports of the disease, other positions have also been reported, including the pineal region, pons, thalamus, spinal cord, optic chiasm, cerebellar hemisphere, optic pathway, lateral ventricle, septum pellucidum, cerebellar vermis, and even temporal lobe [6–9].

With regards to the English literature through a comprehensive search of Web of Science and PubMed using the search term “the rosette-forming glioneuronal tumor” nearly a decade, more than 100 articles have been published to date. After full text screening, excluding articles that were less relevant to the characteristics of RGNTs, nearly 70 articles were included by December 2020 finally. In general, 101 cases of RGNTs were reported located in the fourth ventricle, while 51 cases were located in atypical site. However, the imaging manifestations of RGNTs occurring outside the fourth ventricle are not specific; so, they are often misdiagnosed. Here, the characteristics of RGNTs located outside the fourth ventricle in 51 published cases were listed (Table 1). The lesions can be solid-cystic, cystic, or simple solid, and generally, the former is the most common. The average age of these published cases is 38 years old. Hemorrhage is rare in RGNTs, and only six cases presented positive for bleeding. Management of RGNTs has been accordant with the literatures. Surgery remains the primary treatment option, with gross total resection (GTR) recommended and subtotal resection (STR) as alternatives. The prognosis of RGNT is generally good, and recurrence is uncommon with a total of 4 cases recrudesced of the 51 cases. However, two patients died of these presented cases. Most of the tumors were single lesions with only 10 cases showed multiple lesions.

**Table 1:** Radiological presentation and characters of 51 cohort of patients

Author and year	Lesion	Case number	Age, sex	Location	Contrast enhancement	T1WI	T2WI	Hemorrhage	Management	Recurrence	Number of lesions	Follow-up
Pierre-Aurelien Beuriat et al., 2015	Cystic	1	13/F	Left cerebellar hemisphere	No enhancement	Hypo	Hyper	NA	STR	No	1	NA
Aaron Halfpenny et al., 2019	Cystic	2	5/F	Left temporal lobe	Nodular enhancement	Iso/hypo	Hyper	NA	GTR	Yes,10Y	1	10Y
Lian Duan et al., 2017	Cystic-solid	3	26/F	T9–11	Heterogeneous enhancement	Hypo	Hyper	NA	GTR	No	1	15M’
Lian Duan et al., 2017	Cystic-solid	4	35/F	C3–7	Partchy and inhomogeneous	Hypo	Hyper	NA	GTR	No	1	17M’
Shuji Hamauchi et al., 2019	Cystic-solid	5	37/F	C2–5	Slight enhancement	Hypo	Hyper	NA	GTR	No	1	2Y
Marc Eastin et al., 2016	Cystic	6	33/F	Right thalamic, the ventricle	No enhancement	Hypo	Hyper	NA	GTR	No	2	12M’
Adrien Collin et al., 2018	Cystic-solid	7	40/F	C7–8	Heterogeneous enhancing	Hypo	Hyper	NA	GTR	No	1	6M’
Yazeed Al Krinawe et al., 2020	Solid	8	7/F	Septum pellucidum	No enhancement	Hypo	Hyper	NA	STR	No	1	2Y
Bharadwaj, Rishab et al., 2020	Cystic-solid	9	12/M	The optic pathway	No enhancement	Hypo	Hyper	NA	Biopsy	No	1	6M’
Fumine Tanaka et al., 2019	Cystic-solid	10	18/M	Pons	Partial rim enhancement	Hypo	Hyper	NA	Biopsy	No	1	17Y
Emily P Sieg et al., 2016	Cystic-solid	11	8/F	Right hypothalamus	Ring-like enhancement	Hypo	Hyper	NA	STR	No	1	3Y
ArunkumarSekar et al., 2019	Cystic-solid	12	16/M	Optic chiasm	Ring-like enhancement	Hypo	Hyper	NA	STR	No	1	NA
Goutam Bera et al., 2017	Cystic-solid	14	16/M	Left side of the vermis	Patchy enhancement	Hypo	Hyper	NA	GTR	No	1	1Y
Ji Xiong et al., 2012	Cystic-solid	15	38/M	Septum pellucidum, the bilateral ventricles	Heterogeneous enhancement	Hypo-iso	Mainly hyper	NA	STR	No	2	6M’
Kieren S.J. Allinson,2015	Mainly cystic	16	33/M	The fourth ventricle, the third and lateral ventricles	Patchy enhancement	Hypo	Hyper	NA	Biopsy	Proximately doubled in size	multiple	1Y
Noriko Sumitomo et al., 2017	Cystic	17	9/M	The right parietal lobe	No enhancement	Hypo	Hyper	NA	STR	No	1	NA
Caleb P. Wilson et al., 2020	Cystic-solid	18	19/M	Left temporal, left gangliocapsular region, bilateral thalami, tectum, cerebellum.	Slight enhancement	Hypo	Mildly hype	NA	STR	Dramatic expansion, number increasing	multiple	6Y
L.Gao et al., 2018	Cystic	19	16/M	Cerebellar hemisphere	Slight enhancement	Iso-hypo	Hypo-hyper	NA	GTR	NA	1	NA
L.Gao et al., 2018	Cystic	20	29/M	Lateral ventricle	Heterogeneous enhancement	Iso-hypo	Hypo-hyper	NA	GTR	NA	1	NA
L.Gao et al., 2018	Cystic-solid	21	23/M	Cerebellar vermis	Heterogeneous enhancement	Hypo-hyper	Hypo-hyper	NA	STR	NA	1	NA
L.Gao et al., 2019	Cystic	22	24/M	Left temporal lobe	No enhancement	Hypo	Hyper	NA	GTR	No	1	NA
L.Gao et al., 2019	Cystic	23	30/M	Cerebellar vermis	No enhancement	Hypo	Hyper	NA	GTR	No	1	NA
Sajjad Muhammad et al., 2019	Cystic-solid	24	22/NA	Pineal region	Partially and heterogeneous enhancement	Hypo	Hyper	NA	STR	No	1	8W
Ibrahim Alnaami et al., 2013	Solid	25	57/M	The posterior third ventricle.	Heterogeneous enhancement	Iso	Hyper	NA	Biopsy	No	1	6M’
Ibrahim Alnaami et al., 2013	Cystic-soLid	26	28/M	Posterior third ventricle extending into the aqueduct	Nodular enhancement	Hypo	Hyper	NA	Biopsy	No	1	NA
Özlem Yapıcıer et al., 2018	Cystic	27	55/F	Mesial temporal lobe	No enhancement	Iso-hypo	Heterogeneously hyper	NA	GTR	No	1	NA
H.Cebula et al., 2016	Cystic-solid	28	75/F	Left posterior thalamic	Heterogeneously hyperintense	Heterogeneously hypo	Heterogeneously hyper	Intralesional bleeding	Biopsy	No progression	1	1Y
Sonia García Cabezas et al., 2014	Cystic-solid	29	24/M	Both cerebellar hemispheres, the left cerebellopontine angle, spinal cord	Intense and heterogeneous enhancement	Iso-hypo	Hypo-hyper	NA	Biopsy	No	Multiple	2Y
Shi-Yun Chen et al., 2016	Cystic-solid	30	17/M	Right basal ganglia	Heterogeneous enhancement	Hypo	Hyper	NA	STR	No	1	3Y
Shi-Yun Chen et al., 2017	Cystic	31	33/M	Left parietal lobe	No enhancement	Hypo	Hyper	NA	STR	No	1	3Y
Shi-Yun Chen et al., 2018	Cystic-solid	32	21/F	The third and fourth ventricles and the suprasellar region	Heterogeneous enhancement	Hypo	Hyper	NA	STR	Dead	Multiple	3Y
Yasutaka Fushimi et al., 2011	Cystic-solid	33	28/F	The cerebellar vermis	No enhancement	Hypo	Hyper	NA	STR	No	1	2Y
David Cachia et al., 2014	Cystic	34	36/F	Right frontal lobe, right midbrain tectum, and cerebellar vermis	No enhancement	Hypo	Hyper	NA	STR	No	Multiple	7M’
Philip GeorgeEye et al., 2017	Cystic-solid	35	35/M	The third ventricle	No enhancement	Iso	Hyper	NA	STR	NA	1	NA
Orestes E. Solis et al., 2011	Cystic-solid	36	16/F	The pineal gland region	No enhancement	Iso-hypo	Hyper	NA	STR	No	1	2M’
Ji Xiong et al., 2013	Cystic	37	23/M	Left frontal lobe	No enhancement	Hypo	Hyper	NA	GTR	No	1	8M’
Gorky Medhi et al., 2015	Cystic-solid	38	32/M	Midline posterior fossa, vermis and cerebellar hemispheres	Heterogeneous enhancement	Iso-hypo	Heterogeneously hyper	YES	STR	No	Multiple	11M’
Gorky Medhi et al., 2015	Cystic-solid	39	38/F	Pineal region	Heterogeneous enhancement	Iso-hypo	Heterogeneously hyper	YES	STR	Residual lesions, stable	1	3Y
Gorky Medhi et al., 2015	Cystic-solid	40	24/M	Cerebellar hemispheres, vermis, midbrain, pons, medulla	No enhancement	Iso-hypo	Heterogeneously hyper	YES	STR	Residual lesions	Multiple	NA
Gorky Medhi et al., 2015	Cystic	41	12/M	Pineal region	No enhancement	Hypo	Hyper	NO	GTR	Residual lesions	1	3M’
Gorky Medhi et al., 2015	Cystic-solid	42	40/F	Right cerebellar hemisphere and vermis	Annular and nodular enhancement	Iso-hypo	Heterogeneously hyper	YES	GTR	Minimal residue	Multiple	4M’
S.Kemp et al., 2012	Cystic	43	33/M	Left lateral ventricle	No enhancement	Hypo	Hyper	NA	GTR	NA	1	NA
Ewa Matyja et al., 2014	Cystic	44	22/M	The left temporal lobe.	No enhancement	Hypo	Hyper	NA	GTR	No	1	3.6Y
Junqing Xu et al., 2012	Cystic-solid	45	39/M	Pineal gland, the third ventricle	Faint heterogeneous contrast enhancement	Hypo	Iso-hyper	NA	GTR	No	Multiple	42M’
Benjamin Thurston et al., 2012	Cystic-solid	46	8/F	Left superior cerebellar peduncle	Heterogeneous enhancement	Hypo	Hyper	NA	GTR	Yes, 9M	1	9M’
Anil K. Mahavadi et al., 2020	Cystic-solid	47	41/M	The third ventricle	Heterogeneous enhancement	Mainly hypo	Mainly hyper	NA	STR	Residual lesions, stable	1	6M’
Pankaj Sharma et al., 2011	Cystic-solid	48	16/F	The tectal region of the midbrain	No enhancement	Hypo	Hyper	NA	Biopsy	Stable	1	6M’
Pankaj Sharma et al., 2012	Cystic-solid	49	17/M	Suprasellar and interpeduncular cistern, the third ventricle	Peripheral enhancement	Mainly hypo	Mainly hyper	YES	Biopsy	Stable	Multiple	NA
Seiji Yamada et al., 2019	Cystic-solid	50	16/F	Right temporal lobe	Multinodular enhancement	Hypo	Hyper	NA	GTR	NA	1	NA
Tanmoy Kumar Maiti et al., 2014	Solid	51	2/M	The posterior third ventricle	No enhancement	Hypo	Hyper	NA	STR	Dead	1	13M’
Tanmoy Kumar Maiti et al., 2015	Cystic-solid	52	12/M	The posterior third ventricle	Mild contrast enhancement	Hypo	Hyper	NA	GTR	No	1	9M’

*M* man, *F* female, *NA* not available, *GTR* gross total resection, *STR* subtotal resection, *iso* iso-intensity, *hypo* hypo-intensity, *hyper* hyper-intensity, *Y* year, *M’* month

In summary, the misdiagnosis in the above case reflects how RGNT is under-emphasized and poorly researched. Thus, based on the analysis of the present case and limited data available from review of literature, we propose that the following two aspects may have contributed significantly in misdiagnosing RGNT. Firstly, the case we highlighted occurred in the brain parenchyma, while more than 69.7% of previously reported RGNTs involve the fourth ventricle [3]. And multiple lesions involving the bilateral cerebellar hemisphere, brain stem, and left thalamus at the same time are rarely reported. Secondly, the imaging findings of this case overlapped with cerebral cysticercosis. As we all know, cerebral cysticercosis is the most common parasitic disease of the central nervous system (CNS). The imaging manifestations of the parenchymal active phase are multiple cystic lesions. The enhancement is not obvious and the hypo-perfusion on perfusion imaging. For the above reasons, the tumors were misdiagnosed as cerebral cysticercosis deservedly.

Admittedly, there are distinct radiological signs which highlight uncertainties regarding the previous diagnosis: most notably include tiny spot-like hypo-intense in bilateral cerebellar hemisphere on the susceptibility weighted imaging (SWI) (Fig. 1l). After excluding tiny calcifications on CT (Fig. 1c), it can be assumed that there is minor hemorrhage within the lesions. Hemorrhage is almost invisible in cerebral cysticercosis, although there were also few reports of hemorrhages in RGNT. Two possible reasons may account for the latter. One being that the SWI sequence is rarely a routine sequence, and minor hemorrhages in many reported cases may go undetected because they are difficult to show on other sequences in MRI. On the other hand, it is generally assumed that RGNT is a benign tumor, microvascular proliferation is rare; therefore, the hemorrhages are infrequent as well. The evidence between microvascular endothelial proliferation and hemorrhages has been documented in RGNT [10, 11]. L. Gao et al. reported several cases of RGNTs with intratumoral hemorrhage in 2017. They summarized that intratumoral hemorrhage was one of the additional indications to the diagnosis of RGNT. Other indications included “green bell pepper sign,” CSF dissemination, and multiple satellite lesions. Medhi et al. also summed up that hemorrhage and CSF dissemination may be the characteristics of RGNT through the summary of 7 cases [12]. In our case, intratumoral hemorrhage and multiple satellite lesions are consistent with their conclusions. Therefore, the above signs may aid in diagnosing RGNTs. However, whether this microvascular proliferation and intratumoral hemorrhage are related to prognosis needs further research.

## Conclusion

RGNT is an uncommon low-grade neuroglial tumor which is generally considered benign with slightly longer course [2]. Headache is the most recorded common symptom. Histopathologically, RGNT consists of two components: a neurocytic component that forms rosettes, and an astrocytic component that resembles a pilocytic astrocytoma [13]. Through the case we reported, we have discovered that RGNT can be multiple cystic lesions, and the brain parenchyma can be the major affected areas. The intratumoral hemorrhage shown by SWI sequence may have some significance for our diagnosis of it. In conclusion, RGNT often presents significant diagnostic dilemma, and hence, further knowledge of this tumor is essential as they are relatively slow growing and exhibit benign histological characteristics.
